# Evaluation of Iodine Deficiency in Children with Attention Deficit/Hyperactivity Disorder

**DOI:** 10.4274/jcrpe.2406

**Published:** 2016-03-01

**Authors:** Saliha Kanık Yüksek, Zehra Aycan, Özgür Öner

**Affiliations:** 1 Ankara Children’s Hematology Oncology Training and Research Hospital, Clinic of Pediatrics, Ankara, Turkey; 2 Dr. Sami Ulus Maternity and Children’s Training and Research Hospital, Clinic of Pediatric Endocrinology, Ankara, Turkey; 3 Ankara University Faculty of Medicine, Department of Child and Adolescent Psychiatry, Ankara, Turkey

**Keywords:** iodine deficiency, attention deficit/hyperactivity disorder, children

## Abstract

**Objective:**

To investigate the incidence of iodine deficiency (ID) and its effects on mental function in children referred to the Dr. Sami Ulus Maternity and Children’s Training and Research Hospital with a prospective diagnosis of attention deficit/hyperactivity disorder (ADHD).

**Methods:**

The study was conducted on 89 children referred in the period from September 2009 to June 2010 with a diagnosis of ADHD. A questionnaire was given to all parents. Conners’ rating scales were applied to the parents (CPRS) and teachers (CTRS), and revised Wechsler intelligence scale for children (WISC-R) to the children. Serum thyroid-stimulating hormone, free triiodothyronine and free thyroxine, thyroglobulin, anti-thyroid peroxidase, anti-thyroglobulin, and urinary iodine levels were measured in all children.

**Results:**

Median age was 9.41±1.95 years, and 83.1% of subjects were male. The mean urinary iodine level of the children was 92.56±22.25 μg/L. ID was detected in 71.9% of subjects and all were mild ID. There was no significant relationship between urinary iodine levels with WISC-R subtest scores and CPRS. However, a significant association was found between urinary iodine levels and hyperactivity section of CTRS (p<0.05). Likewise, a significant relationship was found between learning disorder/mental retardation diagnosis and freedom subtest of WISC-R (p<0.05).

**Conclusion:**

This study highlights the effects of ID on comprehension, perception, attention, and learning. However, the results need to be supported by new randomized controlled trials.

**WHAT IS ALREADY KNOWN ON THIS TOPIC?**

Studies in recent years suggested that moderate iodine deficiency (ID) negatively affects the cognitive development even in children with normal thyroid hormones, and may be the cause of decline in school performance. However, the mechanism of cerebral damage due to ID has not been elucidated.

**WHAT THIS STUDY ADDS?**

This study, which has not been made previously in this regard to our knowledge, is important to highlight the effects of ID on comprehension, perception, attention and learning, which are less known and less conspicuous signs of ID. However, the results should be supported by new randomized controlled trials in order to contribute to clinical applications.

## INTRODUCTION

Iodine is an essential element in the synthesis and regulation of thyroid hormones that are required for normal metabolic functions (1). Iodine deficiency (ID) may affect individuals of all ages and is the most common cause of preventable mental retardation, infants being at most risk during the fetal period (2,3). In a state of iodine insufficiency, the synthesis of thyroid hormones is impaired and major health problems ensue. This group of diseases is known as ID disorders (4). While goiter is a well-known symptom of ID disorders, learning, perception, and comprehension disorders also develop in these patients, but these aberrations may at times be difficult to detect in school children and in adults (4,5). The majority of children with ID disorders brought to psychiatric outpatient units by their families or referred to these units by their teachers present with complaints of lack of attention and consequent failure (6). In a substantial portion of these children, attention deficit/hyperactivity disorder (ADHD) needs to be considered in the differential diagnosis (7). ADHD is a disorder in the etiology of which neurological, genetic, environmental, dietary, biological, and psychosocial factors are possibly involved (8,9).

In this present study, we aimed to investigate the incidence of ID in a group of children diagnosed with ADHD and its effect on mental functions such as perception, attention, comprehension, and learning.

## METHODS

Eighty-nine school-age children who were referred to the psychiatric outpatient unit of Dr. Sami Ulus Maternity and Children’s Training and Research Hospital with a diagnosis of ADHD in the time period from September 2009 to June 2010 were prospectively evaluated. Patients with a history of chronic systemic disease, neurological deficit, and chronic drug usage were excluded. None of the patients had a history of identified TSH abnormality by neonatal screening, or a previous history of goiter or an endocrine disease. Ethical approval was obtained for the study as well as verbal and written consent from the families after detailed information was given to them about the subject and purpose of the research. All parents were subjected to a questionnaire which included descriptive information about the child and the family, iodized salt usage status, and the state of awareness of the parents about iodized salt. ADHD and other possible additional disorders such as oppositional defiant disorder (ODD), conduct disorder (CD), and learning disorders/mental retardation (LD/MR) were evaluated according to Diagnostic and Statistical Manual of Mental Disorders-IV criteria (10). At the beginning of the study, both the parents (CPRS) (11) and the teachers (CTRS) (12) of the children were subjected to the Conners’ rating scale in order to examine the developmental processes of ADHD. Revised Wechsler intelligence scale for children (WISC-R) (13), which is the only standardized intelligence assessment test in Turkey, was used to assess the mental condition of the children. The crude scores from the test sections were translated into standard scores according to the age of the children and assessed with improved intelligence quotient (IQ) tables. Anthropometric measurements and physical examination of the thyroid gland were performed in all children.

Serum thyroid-stimulating hormone (TSH), free triiodothyronine (fT_3_) and free thyroxine (fT_4_) (all listed before; chemiluminescence method, Immulite BioDPC, Los Angeles), thyroglobulin (Tg), anti-thyroid peroxidase (anti-TPO), and anti-Tg levels (using the electro-chemiluminometric assay method, Abbott Architect, USA) were determined in venous blood samples in all patients. Urinary iodine levels in a morning spot urine sample were also measured using the (inductively-coupled plasma mass spectrophotometer method). Evaluation of iodine excretion in the urine was performed according to the World Health Organization (WHO) criteria (14) (mild [50-99 μg/L], medium [20-49 μg/L], severe [<20 μg/L], normal [100-200 μg/L], and higher than normal [>200 μg/L]). Thyroid gland palpation was performed in all patients and the results based on WHO goiter staging were recorded (3). Thyroid ultrasonography by an experienced radiologist was performed in all patients in whom goiter had been detected.

### Statistical Analysis

The Statistical Package for the Social Sciences version 16.0 (SPSS Inc., Chicago, IL, USA) was used in the evaluation of the data. Arithmetic average of the values (x), standard deviations (SD), and significance levels (p-values) were shown. Data of the questionnaire specified by count were evaluated as number and percentage. Categorical variables were compared using the chi-square test. Comparison of continuous variables in the groups with and without ID was made by one-way variance analysis. The relationship between rating scores obtained from parents/teachers and the urine iodine levels were assessed by Pearson’s correlation coefficient. A p-value of 0.05 was considered statistically significant.

## RESULTS

### Descriptive Characteristics

The age of children ranged from 6 to 15.5 years (mean 9.41±1.95), and 83.1% (74/89) were male (male:female t-ratio 4.9). Mean height standard deviation score (SDS) of the group was 0.16±1.02 and mean SDS for body weight was 0.07±1.09. The majority of the families were from central and northern parts of Turkey (84.26%). The rate of families living in central Turkey was 89.9%, and 83.1% of them were living in a city center. The majority of parents (44.95%) had completed compulsory basic education. Only 7.85% had completed high school. Most families were of a middle (49.43%) or low (41.57%) socioeconomic status.

### Iodized Salt Usage and State of Consciousness

According to the questionnaire results, 96.6% of the households were using iodized salt and rock salt was consumed by all non-iodized salt users. Salt was stored in a glass lid jar (46.06%) or an opaque lid jar (31.46%). Salt was generally added to the food during cooking (71.91%).

### Goiter, Thyroid Tests and Urine Iodine Levels

Goiter was detected in 5.61% (5/89) of cases and showed different stages (stage Ia in 1 of the 5 cases [ID-related], stage Ib in 2 [Hashimoto thyroiditis in one, normal thyroid volume by ultrasonography in one], and stage II in 2 cases [ID-related]) by palpation. TSH, fT_3_ and fT_4_, Tg, thyroid auto-antibody levels, and urine iodine levels of the patients are given in Table 1. A high TSH level was detected in 2.2% of the subjects. fT_3_ and fT_4_ levels were within normal limits in all subjects. All detected ID cases were in the mild ID group.

In the comparison of the groups with normal iodine levels and those with mild ID, no significant relationship was found with place of residence or of educational level of the families (p>0.05). Also, no correlation was found between ID with salt type used or with time of salt adding to foods (p>0.05).

### Attention Deficit/Hyperactivity Disorder Diagnosis, Revised Wechsler Intelligence Scale for Children Test and Conners’ Scales

The distribution of ADHD subtypes was as follows: ADHD-combined type was seen in 83.1% of patients, ADHD-predominantly inattentive type- in 10.1%, and ADHD-predominantly hyperactive-impulsive type was found in 6.7% of subjects. Rates of additional diagnosis were as follows: only one additional diagnosis in 30.7% of patients, two additional diagnosis in 27.3%, three additional diagnosis in 9.1%, and no additional diagnosis in 32.9%. Anxiety was present in 13/89 (14.6%), LD or MR in 26/89 (29.21%), and ODD/CD in 33/89 (37.07%).

**Figure 1:**
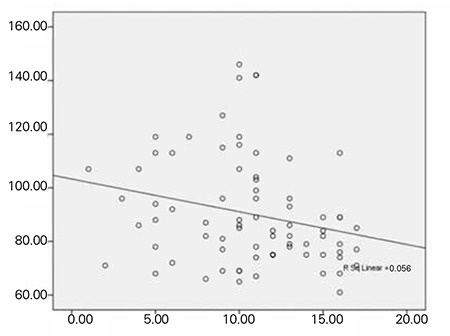
The relationship between hyperactivity section scores (horizontal values) of Conners’ teacher rating scale and urinary iodine levels (vertical values [μg/L])

Mean scores of the WISC-R subtests were as follows: information 6.25±2.72 (1-14), similarities 7.35±3.64 (0-19), arithmetic 7.62±2.66 (1-12), block design 8.60±2.54 (3-18), freedom 23.53±6.04 (8-35), verbal IQ 81.09±14.65 (50-114), performance IQ 88.94±14.53 (51-127), and total score 84.01±13.60 (51-118). Mean scores of CPRS were as follows: hyperactivity (HA) section 8.68±2.82 (3-12), attention deficit (AD) section 7.66±3.04 (1-14), and behavior problems (BP) section 13.67±8.86 (1-52). And mean scores of CTRS were resulted in HA as 10.68±3.88 (1-17), in AD 13.84±4.57 (6-23), and in BP 7.96±4.34 (0-15). Comparison of urinary iodine levels of patients with Conners’ and WISC-R results are given in Table 2. In correlation analysis of the CPRS and CTRS scores with urinary iodine levels, there was a significant negative correlation in HA section of CTRS (p=0.038) with urinary iodine levels (Figure 1), but there was no significant result in other sections. Comparison of freedom section scores of WISC-R and HA section scores of CTRS with the age, gender, and additional diagnosis are shown in Table 3.

## DISCUSSION

Iodine is required in the synthesis and regulation of thyroid hormones and it is known that these hormones have an essential importance for normal metabolic functions. Iodine is an element which should be ingested regularly and in sufficient amounts at all ages. It is not stored in the human body (1,15). About 25% of the world population, including the Turkish population, is faced with ID, a serious public health problem (2). Low intelligence test scores, decrease in intellectual level, deficiency in visual perception and visual motor coordination, speech disorders, incompetence in fine motor skills, balance disorders, isolated deafness, neurological symptoms such as irregular electroencephalogram (EEG) findings were reported in individuals living in ID regions (16,17,18).

Deficiency in intellectual functions varies depending on the time, duration, and degree of exposure to ID. Effects of ID on brain function at various stages of human life has been shown in several studies (4,5). By way of example, the most significant impacts of endemic cretinism on intelligence components are low intelligence and deterioration in visual perception. It has been shown that neurological abnormalities increase and overall intelligence quotients decrease in children exposed to ID, especially in those exposed in the neonatal period (19,20). Studies in recent years suggest that moderate ID negatively affects the cognitive development even in children with normal thyroid hormone levels and may be the cause of decline in school performance (21,22). However, the mechanism of cerebral damage due to ID has not been elucidated.

Tiwari et al (16) investigated the effect of prolonged ID on lack of attention, poor motivation, and learning disorders and reported that while visual learning and memory are affected by severe ID, moderate ID affects motivation more than severe ID does. In another study which aimed to examine the benefits of iodine support in children with euthyroid ID, significant differences in cognitive and motor skills were not detected in repeated tests after iodine supplementation (21). In contrast, improvement in mental function and intelligence scores was reported after iodine supplementation in some similar studies (22,23,24). In an interesting study from Italy, regardless of the iodine levels of the children, ADHD has been claimed to be a syndrome caused by a generalized resistance to thyroid hormone and associated with maternal hypothyroxinemia related to ID in the early gestational period (5). In a systematic review and meta-analysis, technical and methodological limitations of the previous studies on this issue were highlighted, indicating the need for new studies (25). In our study, a significant relationship between ID and verbal performance or performance in other areas of intelligence was not detected. We are aware that lack of a control group is a limitation of our study. New controlled trials including a greater number of patients with moderate or severe ID are needed in order to yield more accurate results. However, we think that the negative correlation we found between urinary iodine levels and HA symptoms requires attention as an important finding in ID-related disorders.

Our study was conducted in an age-compliant target group using a screening method in accordance with WHO recommendations for determining the prevalence of ID (26). Although it is known that the prevalence of goiter is relatively high in school-age Turkish children (27), there are no detailed studies showing the effects of iodine levels on mental functions. Our study was conducted on a limited patient group with disproportionate gender distribution and patient density from the city center. These limitations also need to be considered in the assessment of the study. On the other hand, our study shows that, despite the high rate of iodized salt usage, there is a lack of awareness in Turkish families on the correct storage and usage of iodized salt (2,3). This fact may have caused the high levels of ID in our patients despite the high rate of iodized salt usage. Our results also show that the growth of our patients had not be affected by ID. However, it should be noted that all of our patients detected to have ID were in the mild ID group and short stature is an expected finding in severe ID (3).

The results of our study were similar to reported literature as to sex distribution (28), ADHD subtypes, and additional diagnosis (29,30). Considering the scores of each section of Conners’ rating scales, there is an incompatibility between reported symptoms by the teachers and parents. More precise and accurate observations made by teachers than parents are not surprising because of the low socioeconomic status and low educational levels of the families. Additionally, the results of our study about performance and verbal IQ scores of WISC-R include incompatibility with previous studies (31). However, if the results are analyzed in the light of information that children with ADHD had low scores from the subtests of freedom (the sum of the arithmetic, password and number series sections; also an indicator of distractibility of the attention), cube pattern and image editing of WISC-R, our results are consistent with the literature (32,33).

Unlike other previous studies such as screening of ID in the general population and in school-age children or assessment of the intelligence functions in ID groups, our study aimed to measure actual incidence of ID in lack of attention, comprehension, and perception as well as to assess the effect of ID on learning disorders. This study is important because the effects of ID in children with ADHD have not been examined in previous studies. In our study, a significant negative correlation was detected in HA section of CTRS (p=0.038) with urinary iodine levels. Additionally, statistically significant relationships were determined between age and HA section of CTRS as well as between freedom subtests of WISC-R and additional diagnosis of LD/MR. However, due to the lack of a control group, the confirmation of the detected results is not possible at this stage.

We believe that our results need to be confirmed by randomized controlled studies involving large patient series.

## Ethics

Ethics Committee Approval: Dr. Sami Ulus Maternity and Children’s Training and Researh Hospital Ethics Committee (Approval number: 06.2009/049), Informed Consent: It was taken.

Peer-review: External peer-reviewed.

## Figures and Tables

**Table 1:**
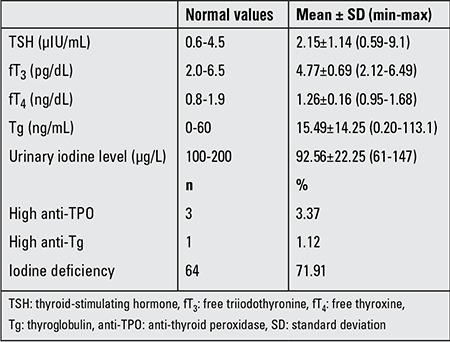
Iodine status of the children (n=89)

**Table 2:**
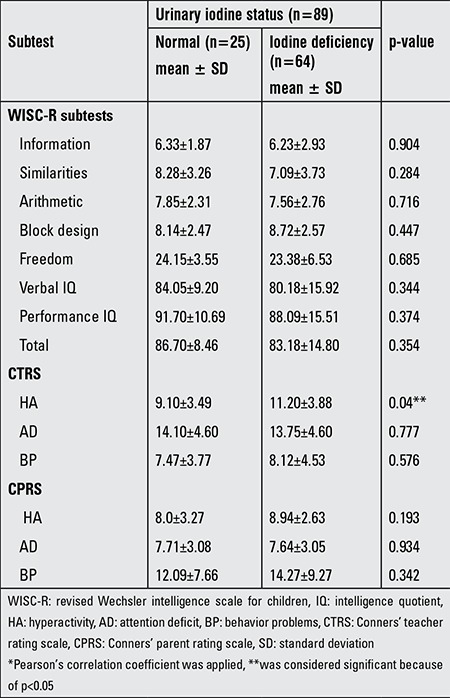
Comparison* of urinary iodine levels with Conners’ scales and revised Wechsler intelligence scale for children results of children

**Table 3:**
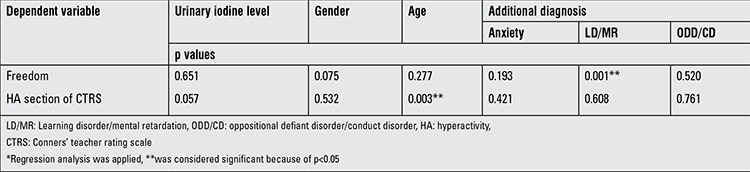
Comparison of freedom section scores of revised Wechsler intelligence scale for children and hyperactivity section scores of Conners’ teacher rating scale with the age, gender and additional diagnosis of children (n=89)*
